# The rise of Mendelian randomization for exploring disease causality: A bibliometric analysis based on CiteSpace

**DOI:** 10.1097/MD.0000000000043764

**Published:** 2025-08-08

**Authors:** Kunyang He, Xiaochu Wu, Zhengyu Qian, Kaijie Lin, Yue Wang, Zhikun Su, Tianyao Zhang

**Affiliations:** a School of Clinical Medicine, Chengdu Medical College, Chengdu, Sichuan Province, China; b The First Affiliated Hospital Of Chengdu Medical College, Chengdu, Sichuan Province, China; c National Clinical Research Center for Geriatrics, West China Hospital, Sichuan University, Chengdu, Sichuan Province, China.

**Keywords:** CiteSpace, differential analysis, highly cited papers analysis, Mendelian randomization research, visualization

## Abstract

**Objective:**

Mendelian randomization (MR) utilizes genetic variants as instrumental variables to explore causal associations between exposures and outcomes. Despite a significant increase in MR-related publication volume in recent years, accompanying bibliometric analyses are lacking. Hence, our study aims to conduct a bibliometric analysis of MR articles published within the last 20 years to elucidate the current research landscape and identify potential future directions.

**Methods:**

We searched the Web of Science Core Collection for articles related to MR from 2003 to 2023. The analysis utilized CiteSpace 6.1.R6 and Excel to examine annual publication volume, keywords, journals, countries, institutions, authors, co-cited references, and highly cited articles.

**Results:**

A total of 7801 articles in the MR field were retrieved between 2003 and 2023, demonstrating a rapid growth in annual publication volume. “INT J EPIDEMIOL” not only possesses the largest academic output but also boasts the highest co-citation. England contributed the largest number of papers, and the Univ Bristol topped the list with the most articles. George Davey Smith is the most prolific author. The article from Hemani G et al (2018) had the most co-citations. England, the Univ Bristol, and Smith, GD played pivotal roles in highly cited articles. Research hotspots primarily encompassed cardiovascular diseases, metabolic diseases, biomarkers, inflammation, Alzheimer disease, cancer, age. Emerging research frontiers in the last 3 years featured obstructive sleep apnea, psychiatric disorders, major depressive disorder, and coffee consumption.

**Conclusions:**

This study has reviewed the research status and trends in the MR field from 2003 to 2023. These findings provide invaluable insights and guidance for the future development of the MR field.

## 1. Introduction

Epidemiological observational studies are often susceptible to confounding, reverse causation, and various biases, often resulting in inconclusive outcomes in attempts to demonstrate the causal impact of modifiable exposure factors on disease outcomes.^[1]^ Furthermore, the conduct of long-term randomized controlled trials (RCTs) with disease outcomes is frequently deemed impractical due to ethical constraints and inadequate compliance.^[2]^ Mendelian randomization (MR) utilizes genetic variations as instrumental variables in observational epidemiology to deduce causal relationships between exposure factors and complex disease outcomes.^[3]^ Compared to traditional RCTs, MR presents several advantages. This method enables control over non-genetic environmental confounders in the analysis and assesses the impact of exposure on outcomes using genetic variations, eliminating the need to collect exposure data in the outcome group. Moreover, as genetic variations typically precede exposure and are not affected by outcomes, MR is able to reduce the influence of reverse causality.

In recent years, MR has seen extensive utilization across various disciplines, including genetics, epidemiology, biomedicine, cardiovascular diseases, oncology, metabolic syndrome, and psychiatric disorders. Bibliometric analysis represents a research method based on literature data. The primary aim is to evaluate the output and influence of academic research by statistically analyzing indicators such as the quantity, quality, and impact of literature. Given the exponential surge in MR-related publications from 2020 to 2023, an updated bibliometric analysis in this domain is necessitated. CiteSpace constitutes a scientific software tool developed using the Java language. This tool is designed to detect and visualize the current state of scientific knowledge, analyze trends in literature development, and pinpoint prospective research directions.^[4]^ This study aims to conduct a bibliometric analysis of MR-related literature from 2003 to 2023 using CiteSpace6.1R4 software, to assess the current research status in this field. Additionally, this analysis involves comparing the distinctions in publication characteristics between 2003 and 2020 and 2003 and 2023, aiming to identify future research directions.

## 2. Methods

### 2.1. Inclusion and exclusion criteria

For the bibliometric analysis in this study, we searched the Web of Science database on July 8, 2023, and the article sources were confined to the Web of Science Core Collection. The search query utilized was TS = (Mendelian Randomization), with a time span ranging from January 1, 2003, to July 8, 2023. The publication type was not restricted; however, only articles in English were included. Following the export of literature from the database, each article’s title and abstract were manually reviewed, excluding those not pertinent to the research topic and duplicates. This selection process aimed to ensure the study’s reliability and validity. Ultimately, 7081 valid articles were retained, comprising 5534 articles and 665 review papers.

### 2.2. Data processing and parameter settings

*Export of references*: In compliance with the predetermined inclusion and exclusion criteria, the chosen literature was exported in plain text format, capturing the full records, and cited references’ content. The exported text files were labeled “download” and stored in a previously created “input” folder. Subsequently, the “Remove Duplicates” feature in CiteSpace was employed to deduplicate the data, thereby enhancing its precision and efficiency. Ultimately, a new project folder in CiteSpace titled “last MR research” was established, and the relevant data was imported.

*Parameter settings*: Following the guidelines from Chen,^[5]^ the following parameters were configured for the relevant analysis in CiteSpace: the time span was established from January 2003 to December 2023, with a time slice of 1 per year. The term source was configured to encompass Title, Abstract, Author, Keywords, and Keywords Plus, ensuring comprehensive results. Pruning was set to Pathfinder, employing a pathfinding algorithm to refine the research network, thereby streamlining the overarching structure and facilitating smooth exploration.^[4]^ The Top N parameter was set at 50, denoting the inclusion of the top 50 most frequently cited references in each time slice, covering a wide range of research topics and disciplines. The k-value was established at 5, while all other parameters were maintained at their default settings.

## 3. Materials and methods

### 3.1. Publication year trends

**Figure 1.**
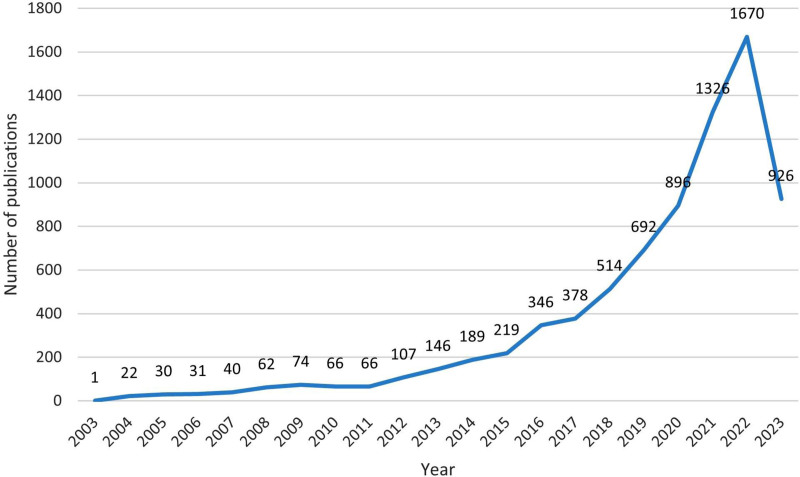
Number of publications in Mendelian randomization in the last 20 years. Each numerical value on the line corresponds to the publication count for the respective year.

Of the 7801 pertinent studies, 5534 were research articles (70.94%), 665 were review articles (8.52%), and 234 were letters (3.00%). Figure 1 delineates the publication trajectory in the realm of MR research. The ensuing evolution can be divided into 3 phases: the initial stage spanning 2003 to 2011 (n = 392), the subsequent stage from 2012 to 2017 (n = 1385), and the final stage from 2018 to 2022 (n = 5098). The period from 2003 to 2011 was characterized by gradual expansion, with publications increasing from a single one in 2003 to 66 in 2011. The span between 2012 and 2017 witnessed rapid growth, as publications surged from 107 in 2012 to 378 in 2017, averaging an annual increment of 54 papers. The period from 2018 to 2022 experienced substantial growth, with publications increasing from 514 in 2018 to 1670 in 2022, indicating an average annual increase of 289 papers. Notably, the growth rate from 2020 to 2021 peaked at 430, marking the highest point in recorded history. Through exponential fitting, a significant correlation was observed between the publication year and the volume of papers, as indicated by a determination coefficient *R*^2^ = 0.8851. Projections suggest that the total number of publications in 2023 is expected to reach 2272.

### 3.2. Keyword co-occurrence analysis

**Figure 2.**
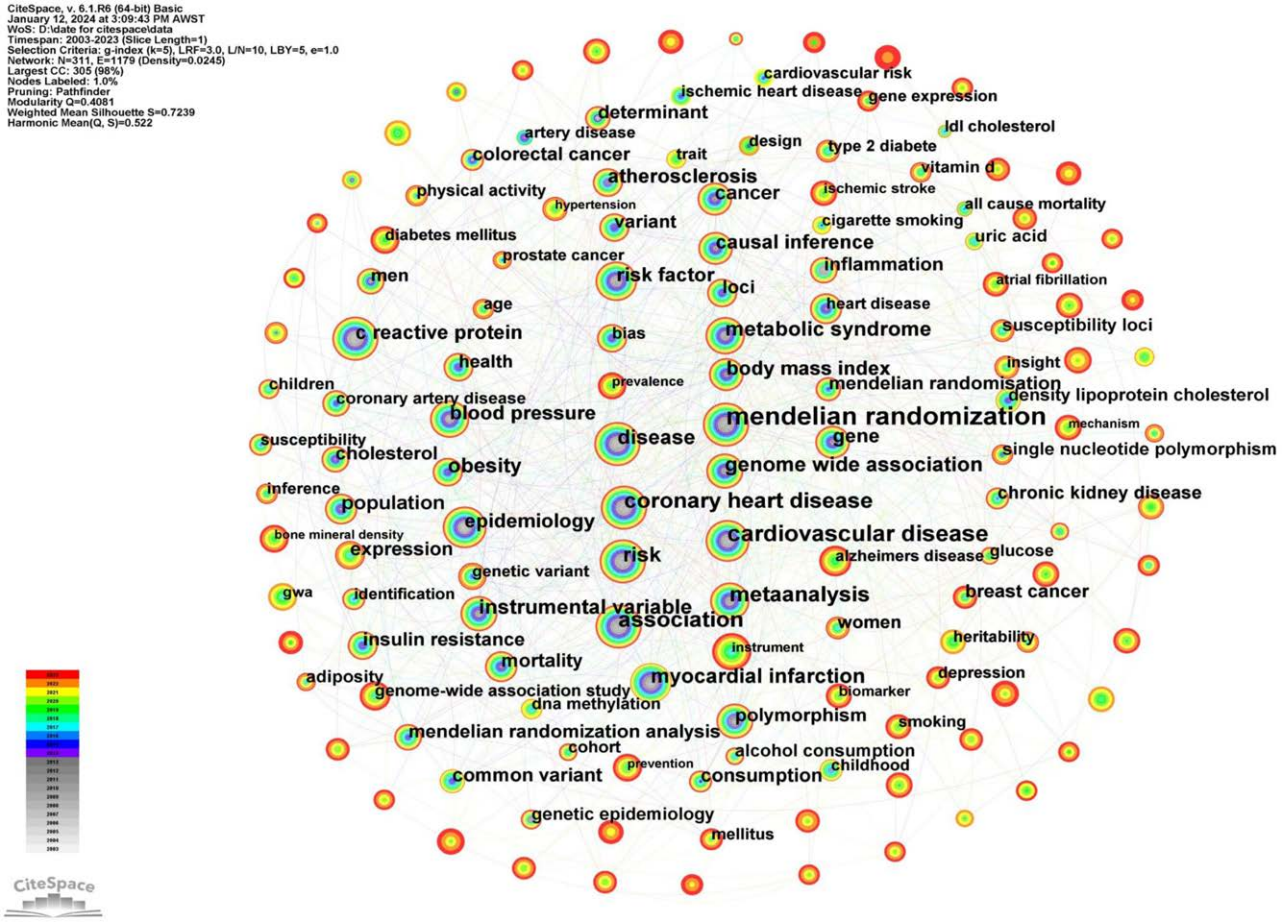
The keyword network map of MR from 2003 to 2023. The visual representation of keyword co-occurrence outcomes, where nodes symbolize keywords. The magnitude of the nodes represents the frequency of keyword appearances, and the links signify co-occurrence associations between keywords. MR = Mendelian randomization.

Keyword co-occurrence analysis can aid researchers in the domain of MR studies by pinpointing research hotspots and trends, offering a snapshot of topics and research territories.^[6]^ In CiteSpace, we set the k-value to 5 and utilized the Pathfinder algorithm to streamline the research network, thus making the co-occurrence map clearer in illustrating keyword interconnections. Figure 2 illustrates the visual representation of keyword co-occurrence outcomes, where nodes symbolize keywords. The size of the nodes indicates the frequency of keyword occurrences, and the edges represent co-occurrence relationships between keywords. The keyword co-occurrence graph includes 311 nodes and 1179 edges, demonstrating a network density of 0.0245. Table 1 lists the top 20 keywords, following the exclusion of those related to methods and techniques in MR research.

**Table 1:** The top 20 keywords in Mendelian randomization from 2003 to 2023.

Rank	Frequency	Centrality	Keywords
1	804	0.13	Cardiovascular disease
2	591	0.04	BMI
3	535	0.09	Coronary heart disease
4	490	0.06	Obesity
5	416	0.06	Blood pressure
6	347	0.03	Mortality
7	289	0.02	Coronary artery disease
8	261	0.05	Insulin resistance
9	258	0.08	Myocardial infarction
10	243	0.07	C-reactive protein
11	238	0.09	Metabolic syndrome
12	220	0.06	Inflammation
13	202	0.02	Heart disease
14	184	0.02	Alzheimer disease
15	171	0.03	Vitamin D
16	168	0.02	Type 2 diabetes
17	162	0.05	Cancer
18	146	0.03	Density lipoprotein cholesterol
19	131	0.03	Age
20	128	0.01	Atrial fibrillation

The top 20 Mendelian randomization research keywords from 2003 to 2023 by frequency, including their ranking, occurrence frequency, centrality, and the keywords.

### 3.3. Keywords burst detection analysis

The burst detection analysis of keywords in CiteSpace is a visual tool that identifies terms with bursts extracted from titles, abstracts, descriptors, and identifiers of literature records. This facilitates the pinpointing of current research frontiers and aids researchers in understanding research hotspots, trends, and development trajectories in a specific domain.^[7]^

**Figure 3.**
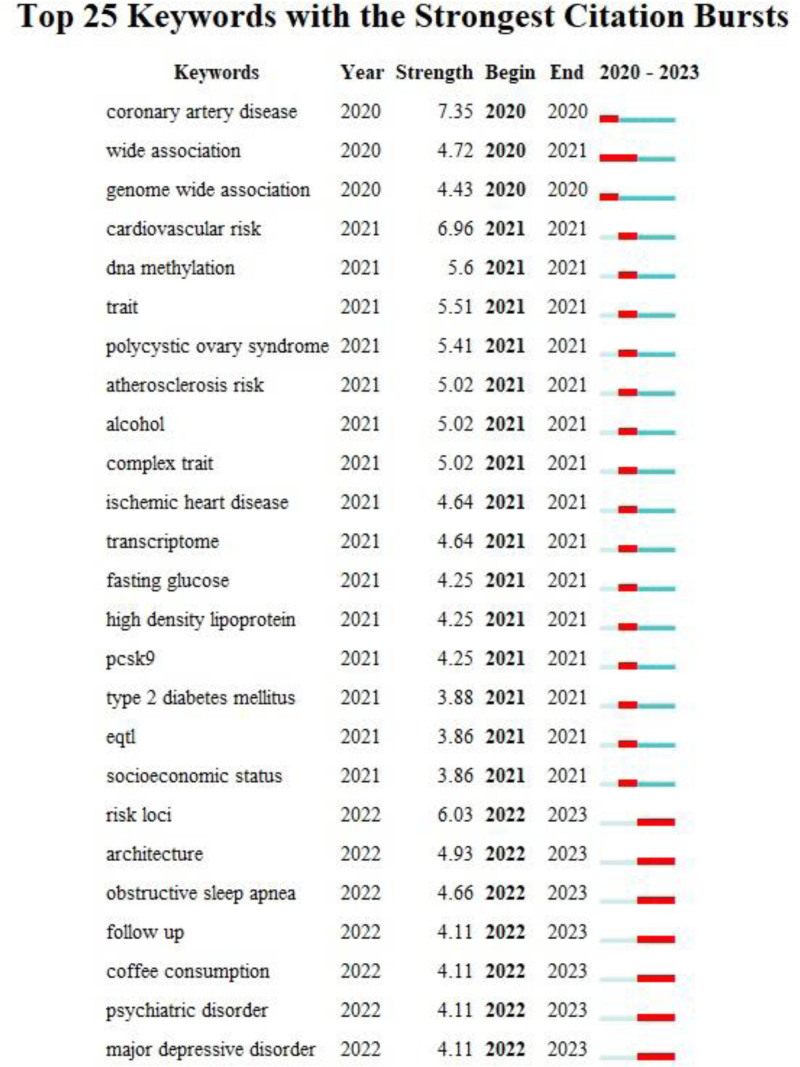
Top 25 keywords with the strongest citation bursts from 2003 to 2023. Top 25 keywords with the strongest citation bursts from 2003 to 2023. Time intervals are delineated by green lines and the spans of burst keywords are accentuated in red.

To better identify the frontier trends in the research field, we have set the time slice for keyword emergence analysis study from 2020 to 2023. Figure 3 showcases the burst keywords as detected by CiteSpace, where time intervals are delineated by green lines and the spans of burst keywords are accentuated in red. After omitting keywords tied to MR research methods, the burst keywords spanning 2022 to 2023 encompass obstructive sleep apnea (OSA), coffee consumption, psychiatric disorder, and major depressive disorder, all of which delineate the prevailing research frontiers in the MR field.

### 3.4. Keyword clustering analysis

**Figure 4.**
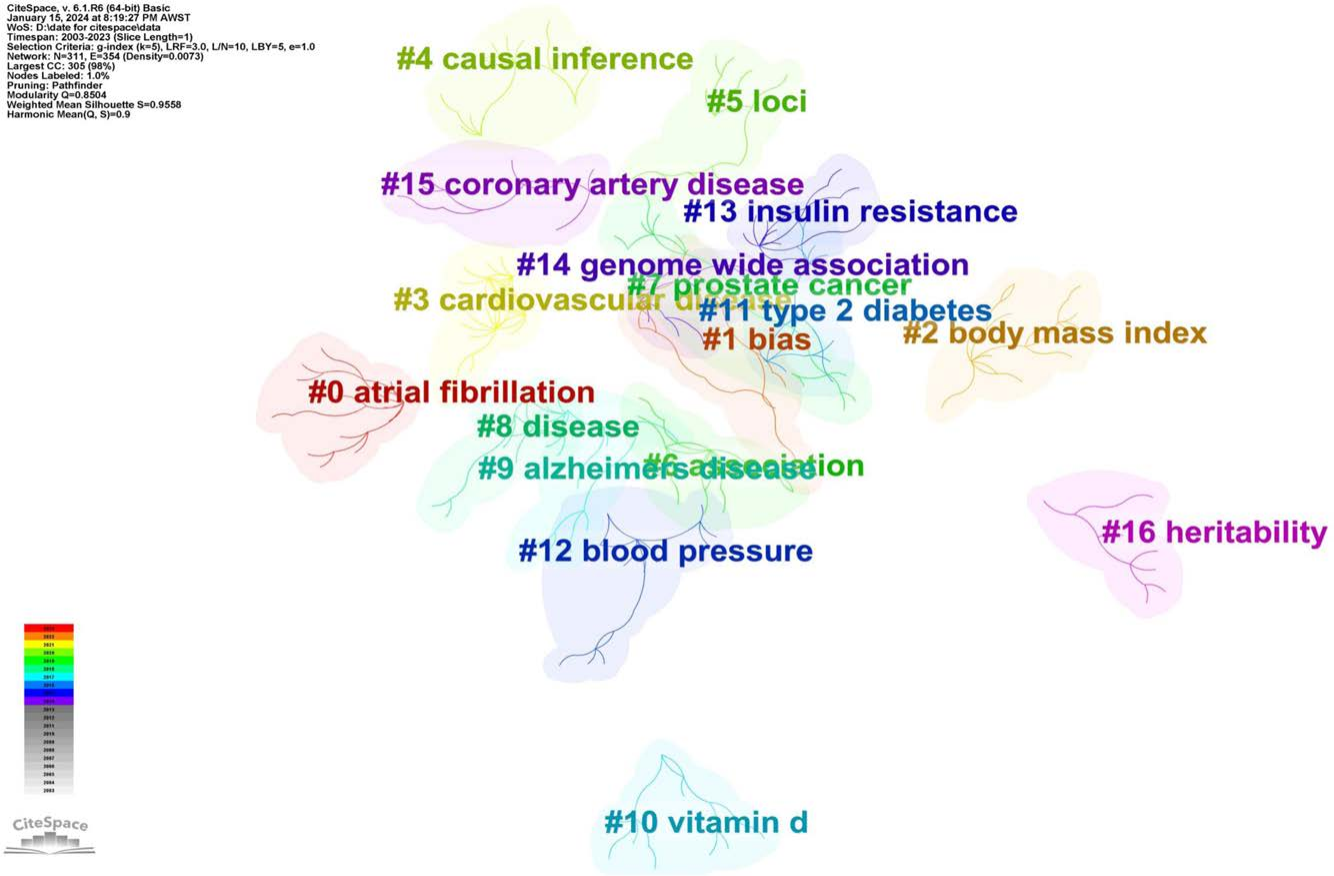
Keyword Cluster Map for MR Studies (2003–2023). A total of 16 distinct clusters have been identified. Overlapping of multiple clusters in the cluster map might be due to the presence of similar or intersecting topics. The depicted values, Q = 0.8504 and S = 0.9558, demonstrate notable statistical significance and robust cluster consistency. MR = Mendelian randomization.

Employing CiteSpace and the LLR algorithm, we conducted a keyword clustering analysis of the included keywords. A total of 16 clusters were identified (Fig. 4, Table 2). Overlaps within the cluster map may occur due to the presence of similar or intersecting topics. The Q-value, representing the cluster module value, offers insights: A Q-value between 0 and 1 indicates the degree of relatedness of keywords within a cluster, with values approaching 1 signifying increased cohesion. A Q-value >0.3 signifies a significant cluster structure. The S value, representing the cluster’s average silhouette value, provides an overview of the relative quality of clustering and data configuration. Ideally, the silhouette value should range between -1 and 1, with values closer to 1 indicating superior consistency. Clusters with an S value >0.5 are considered acceptable, while those with an S value >0.7 are regarded as compelling. Our analysis yielded a Q-value of 0.8504 and an S value of 0.9558, indicating notably significant and coherent clusters. From the keyword clustering analysis, the primary exposure factors and outcomes in MR research include atrial fibrillation, body mass index (BMI), cardiovascular disease, prostate cancer, Alzheimer disease (AD), vitamin D, type 2 diabetes, blood pressure, insulin resistance, and coronary artery disease.

**Table 2:** Clusters of keywords for Mendelian randomization studies from 2003 to 2023.

Clusters ID	Silhouette	Cluster labels	Main keywords
#0	0.987	Atrial fibrillation	Ischemic stroke; heart failure.
#1	0.929	Bias	Variant; Mendelian randomization analysis; GWA; alcohol consumption.
#2	0.944	Body mass index	Obesity; weight; overweight; FTO gene (fat mass and obesity-associated gene).
#3	0.989	Cardiovascular disease	Coronary heart disease; myocardial infarction; causal association; Mendelian randomization.
#4	0.963	Causal inference	Instrumental variable; instrumental variables; genetic variant; identification.
#5	0.942	Loci	Gene expression; genetic epidemiology; plasma; cholesterol.
#6	0.964	Association	Risk; meta analysis; inflammatory bowel disease; ulcerative colitis.
#7	0.927	Prostate cancer	Prostate cancer; breast cancer; genome-wide association study; rheumatoid arthritis; susceptibility loci.
#8	0.972	Disease	Health; epidemiology; cardiovascular disease; Mendelian randomization study.
#9	0.937	Alzheimer disease	Parkinson disease; neurodegenerative diseases; single nucleotide polymorphism; dementia.
#10	0.973	Vitamin D	Bone mineral density; osteoporosis; supplementation; D deficiency.
#11	0.895	Type 2 diabetes	Birth weight, Mendelian randomization; UK Biobank; physical activity.
#12	0.946	Blood pressure	Hypertension; mortality; cardiovascular risk factors; LDL cholesterol.
#13	1.000	Insulin resistance	Metabolic syndrome; diabetes mellitus; risk factor; protein.
#14	0.985	Genome wide association	Common variant; lung cancer; C-reactive protein; causal relationship.
#15	0.935	Coronary artery disease	Ischemic heart disease; heart disease; density lipoprotein cholesterol; atherosclerosis.
#16	0.962	Heritability	LD score regression; insight; trait; common.

The clusters of keywords for Mendelian randomization studies conducted from 2003 to 2023. The clusters are identified by Cluster ID, Silhouette score, Cluster labels, and the Main keywords associated with each cluster. These clusters provide insights into the key research themes within the MR studies during this period.

### 3.5. Journal and co-cited journal analysis

#### 3.5.1. Journal analysis

A total of 7081 articles related to MR were published across 1235 academic journals from 2003 to 2023. Among these, 17 journals published more than 80 articles each, cumulatively accounting for 28.32% of publications. Table 3 details the top 10 academic journals in terms of article publications in the MR realm. “INTERNATIONAL JOURNAL OF EPIDEMIOLOGY” led with the highest number of published articles (n = 292, IF = 7.7, Q1), followed by “FRONTIERS IN GENETICS” (n = 235, IF = 3.7, Q2), “SCIENTIFIC REPORTS” (n = 170, IF = 4.6, Q2), “NATURE COMMUNICATIONS” (n = 132, IF = 16.6, Q1), and “GENETIC EPIDEMIOLOGY” (n = 120, IF = 2.1, Q3). The Impact Factor of a journal denotes the average number of citations its articles receive over recent years. This metric is commonly used as a gauge to assess the influence of a journal within its particular academic domain.^[8]^ Among the top 10 journals, the average Impact Factor stands at 9.87, with 7 journals boasting an Impact Factor exceeding 5.0. These journals collectively published 962 articles, roughly 14% of the overall count.

**Table 3:** Top active 10 journals that published Mendelian randomization-related articles from 2003 to 2023.

Ranking	Journal	n	2022 IF	Quartile	Country
1	International Journal Of Epidemiology	292	7.70	Q1	United Kingdom
2	Frontiers In Genetics	235	3.70	Q2	Switzerland
3	Scientific Reports	170	4.60	Q2	United Kingdom
4	Nature Communications	132	16.6	Q1	United Kingdom
5	Genetic Epidemiology	120	2.10	Q3	International Journal
6	Journal Of Clinical Endocrinology Metabolism	118	5.80	Q1	United States
7	Nutrients	109	5.90	Q1	Switzerland
8	Frontiers In Endocrinology	106	5.20	Q1	Switzerland
9	Circulation	104	37.8	Q1	United States
10	BMC Medicine	95	9.30	Q1	United Kingdom

An overview of the top 10 journals actively involved in publishing articles related to MR research from 2003 to 2023. The ranking is based on the number of articles published in each journal during this period. Additionally, key information such as the 2022 Impact Factor, Journal Quartile, and the journal’s country of origin is included.

#### 3.5.2. Co-cited journal analysis

**Figure 5.**
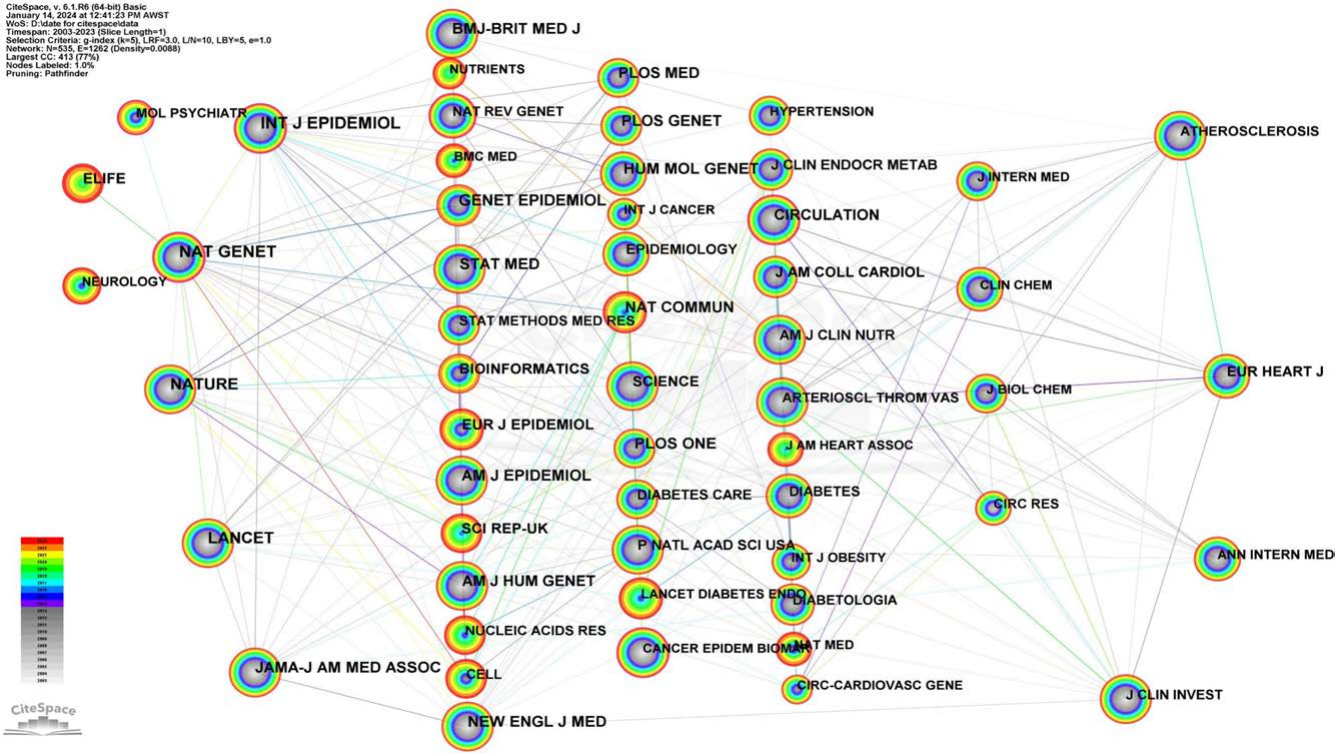
The network map of co-cited journals from 2003 to 2023. The co-cited journal network was constructed using journals with a co-cited frequency ≥ 100 times, Node size represents the co-cited frequency, while links indicate shared co-citation relationships among journals.

The primary objective of using CiteSpace for co-cited journal analysis is to assess the co-citation relationships among academic literature, highlighting journals with substantial impact and elevated citation frequencies. Based on the visualization results (Table 4), we identified the top 10 co-cited journals: INT J EPIDEMIOL, NAT GENET, LANCET, NATURE, GENET EPIDEMIOL. These journals have garnered citation frequencies surpassing 3000 times, boasting an average impact factor of 54.86. Except for GENET EPIDEMIOL, which hails from the United States, the remaining 4 are based in the UK. We constructed a co-cited journal network highlighting journals with a co-cited frequency of 600 or more times (Fig. 5). INT J EPIDEMIOL represents the most substantial node, indicating the highest citation frequency. We identified 2 co-cited journal clusters. The first co-citation cluster primarily comprises INT J EPIDEMIOL (n = 5359, IF = 7.7), NAT GENET (n = 5176, IF = 30.8), LANCET (n = 3559, IF = 168.9), JAMA-J AM MED ASSOC (n = 2921, IF = 120.7), among others. The second co-citation cluster primarily consists of ATHEROSCLEROSIS (n = 1023, IF = 5.2), EUR HEART J (n = 1515, IF = 39.3), ANN INTERN MED (n = 784, IF = 39.2), J CLIN INVEST (n = 864, IF = 15.9), and others.

**Table 4:** The top 10 co-cited journals by frequency from 2003 to 2023.

Rank	Co-cited journals	Frequency	IF
1	Int J Epidemiol	5359	7.7
2	Nat Genet	5176	30.8
3	LANCET	3559	168.9
4	Nature	3517	64.8
5	Genet Epidemiol	3126	2.1
6	Nat Commun	2962	16.6
7	Plos One	2925	3.7
8	JAMA-J Am Med Assoc	2921	120.7
9	Hum Mol Genet	2919	3.5
10	BMJ-Brit Med J	2797	107.7

The top 10 co-cited journals, determined by their co-citation frequency within MR research articles from 2003 to 2023. The ranking is based on the number of times these journals were co-cited during this period. The table also provides the Impact Factor of each journal for reference.

**Figure 6.**
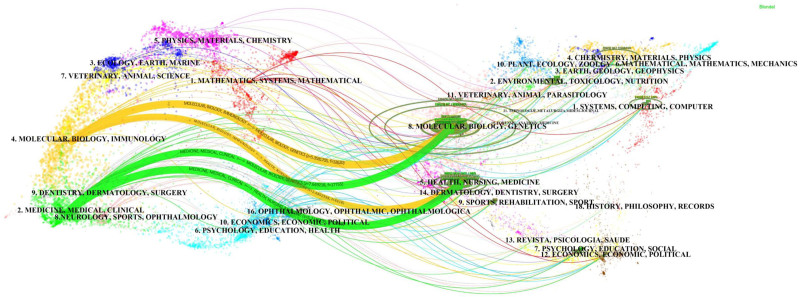
The dual-map overlay of journals related to MR research from 2003 to 2023. The left side represents the citing base map, including citing journals, while the right side represents the cited base map, containing cited journals. Each spline curve originates from a citing journal on the left base map and points to a cited journal on the right base map. The labels indicate the corresponding disciplines in which the citing articles were published, and each label is positioned at the centroid of the corresponding journal cluster. MR = Mendelian randomization.

Figure 6 displays the dual-map overlay of journals. The dual-map concurrently presents the citing and cited coverage maps, reflecting the dynamic progress in the field of MR at the disciplinary level, encompassing citation trajectories, knowledge flow, and the distribution of papers across various information fields.^[9]^ We utilized the Z-score to adjust the clustering graphical interface, aiming for standardization and simplification. The left side represents the citation base map, and the right side represents the cited base map. Journals featured in both the citation and cited base maps are marked with circles, their colors indicating membership in Blondel clusters. The curves represent citation trajectories, originating from the citing journals on the left base map and leading to the cited journals on the right base map. Cluster labels are represented by terms derived from the titles of journals within each cluster, and these labels are positioned at the clusters’ centroids.^[10,11]^ In the left-side diagram, the longer the horizontal axis of the ellipse, the more papers are published in the corresponding journal; the longer the horizontal axis of the ellipse, the more authors are involved.^[12]^ A shift in the citation trajectory from one area to another on the citation journal base map indicates a change in the main disciplines of the related articles, signifying that articles in various disciplines are published in different journals.^[10]^ From the analysis, the upper green trajectory connects “medicine, medical, clinical” in the citing journal base map to “molecular, biology, genetics” in the cited journal base map. The lower green trajectory represents citations from “medicine, medical, clinical” to “health, nursing, and medicine” in the cited base map. The upper yellow trajectory connects “molecular, biology, immunology” in the citing journal base map to “molecular, biology, genetics.” The lower yellow trajectory represents citations from “molecular, biology, immunology” to “health, nursing, medicine.”

### 3.6. Country/region/institution analysis

**Figure 7.**
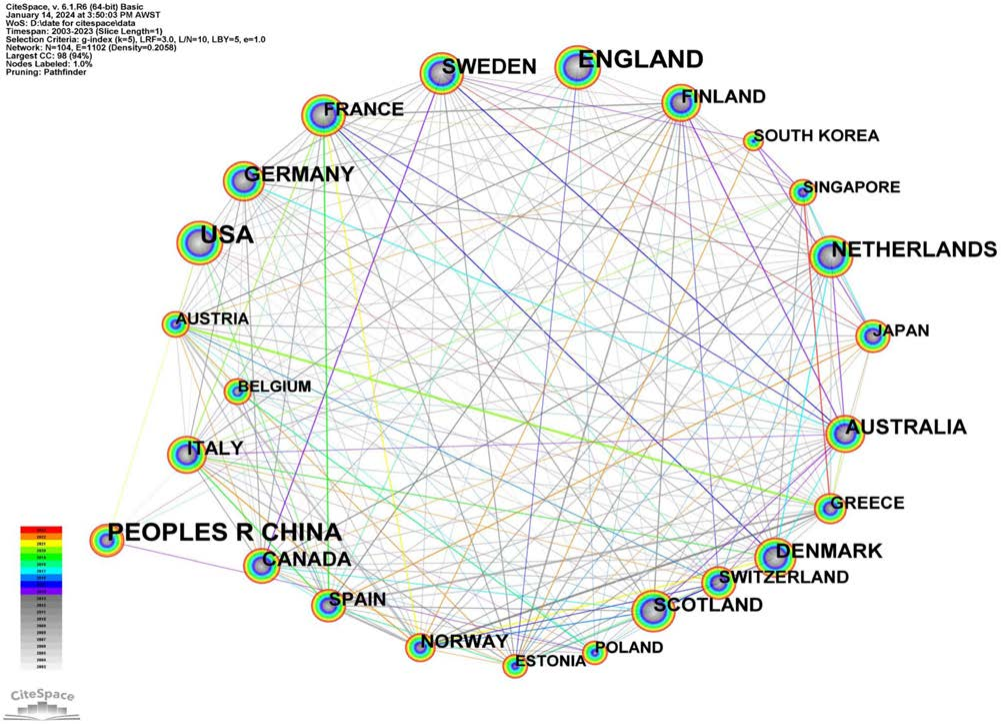
The network map of country collaborations in MR research from 2003 to 2023. The node size represents the number of publications contributed by each country in the field of MR research, and the links indicate collaborations between countries. MR = Mendelian randomization.

A total of 104 countries/regions produced literature related to MR between January 2003 and July 2023. Table 5 showcases the top 10 countries/regions based on their publication count. Notably, 3 countries generated more than 2000 papers each, while 10 countries each produced at least 300 papers. Leading the chart, ENGLAND has the most publications (n = 2550), trailed by the CHINA (n = 2543) and USA (n = 2537). We sculpted a collaboration network featuring countries that contributed over 100 papers (Fig. 7). Here, the node size corresponds to the volume of publications a country has rendered in MR research, while the links elucidate collaborative relationships between nations. Gleaning insights from the Figure 7, prominent nodes like ENGLAND, CHINA, USA, and NETHERLANDS underscore their pivotal research contributions and prowess in the MR arena. Additionally, multiple countries display tight-knit collaborations. For instance, the United States maintains robust cooperative ties with CANADA (n = 529), ENGLAND (n = 2550), SWEDEN (n = 739), and NETHERLANDS (n = 758), among others. Interestingly, while China ranks second in publication volume (n = 2543), has less collaboration with other countries, with partnerships only with Germany, the United Kingdom, South Korea, Singapore, and Poland.

**Table 5:** Ranking of countries in Mendelian randomization research articles from 2003 to 2023.

Ranking	Country	Frequency
1	England	2550
2	Peoples R China	2543
3	USA	2537
4	Netherlands	758
5	Sweden	739
6	Germany	725
7	Australia	654
8	Denmark	558
9	Canada	529
10	Scotland	397

The ranking of nations according to the frequency of their contributions to MR research articles from 2003 to 2023.

**Figure 8.**
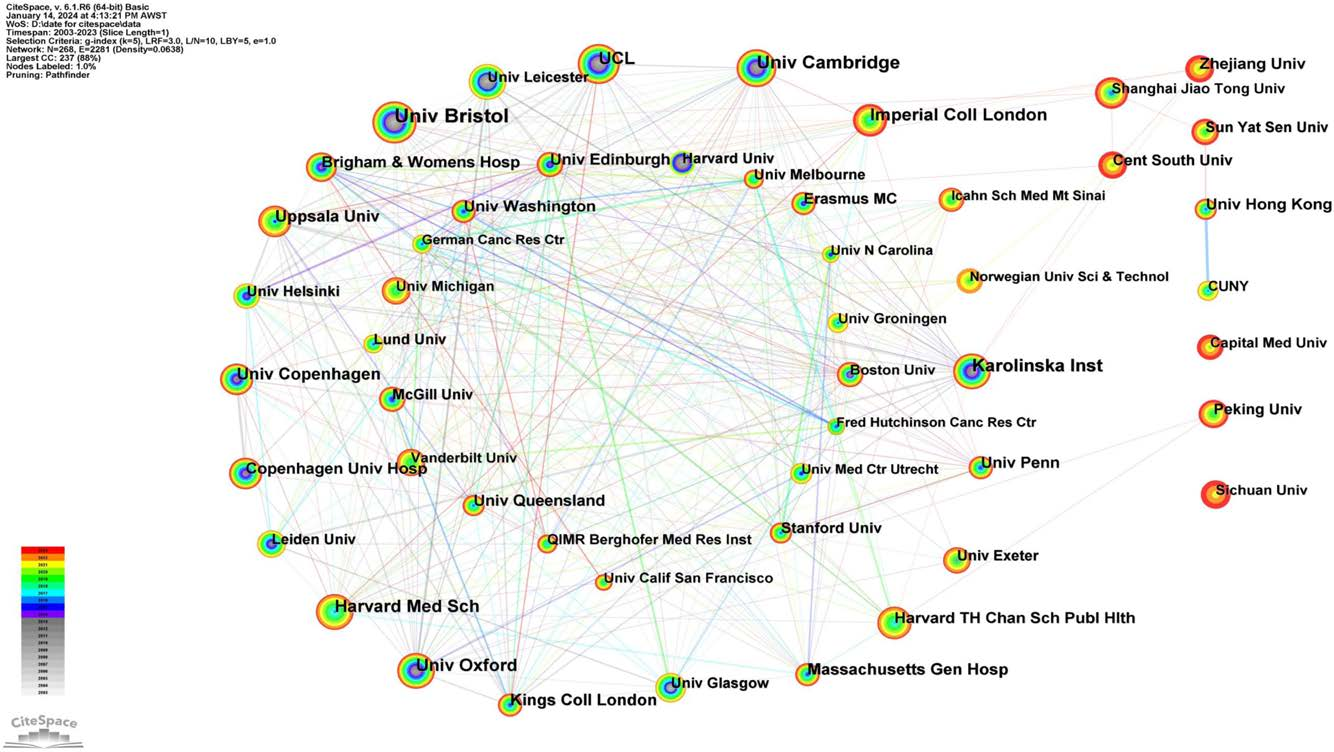
The network map of institution collaborations in MR research from 2003 to 2023. Nodes represent institutions, and node size corresponds to the number of publications contributed by each institution, while the links represent collaborative relationships between institution. MR = Mendelian randomization.

A total of 266 institutions contributed to the publication of MR literature, and Table 6 identifies the top 10 institutions based on their publication count. Of these, 6 institutions are based in the UK, 1 in Denmark, 1 in Sweden, and 2 in the United States. These top 10 institutions have collectively produced 4676 articles, accounting for 60% of the total publications. At the forefront, the University of Bristol leads with the highest number of publications (n = 1084), followed by the University of Cambridge (n = 629), Karolinska Institute (n = 456), the University of Oxford (n = 442), and Harvard Medical School (n = 410). We constructed a collaboration network featuring institutions that contributed over 100 papers (Fig. 8). Here, nodes represent institutions, with the node size reflecting the volume of publications an institution has contributed. The links illustrate collaborative ties, and changes in color represent the research activity levels of various institutions over time. Notably, the node representing the University of Bristol is the most prominent, reflecting its leading publication count. The network reveals rich collaborative dynamics among institutions, including a cooperative circle centered around the University of Cambridge. This circle comprises UCL, Karolinska Institute, the University of Copenhagen, the University of Bristol, Harvard Medical School, the University of Oxford, Imperial College London, Uppsala University, and the University of Pennsylvania, coming from the United Kingdom, Sweden, Australia, and the United States. We also observed that some institutions demonstrate reduced activity in international collaboration, including Sichuan University, Capital Medical University, Zhejiang University, Sun Yat-sen University, the University of Hong Kong, CUNY, Shanghai Jiao Tong University, and Central South University. These institutions exhibit significantly fewer international collaborations compared to others, and most of these less collaborative institutions are situated in China. Additionally, an analysis of the node color changes in the collaboration network diagram reveals that Sichuan University, Capital Medical University, Zhejiang University, Sun Yat-sen University, Shanghai Jiao Tong University, Central South University, and Imperial College London have significantly outperformed other institutions in MR paper output in the past 3 years. Notably, a majority of these high-output institutions are located in China.

**Table 6:** Ranking of institutions in Mendelian randomization research articles from 2003 to 2023.

Ranking	Institution (Country)	Frequency	Centrality
1	Univ Bristol (UK)	1084	0.12
2	Univ Cambridge (UK)	629	0.09
3	Karolinska Inst (Sweden)	456	0.12
4	Univ Oxford (UK)	442	0.21
5	Harvard Med Sch (USA)	410	0.02
6	UCL (UK)	400	0.05
7	Imperial Coll London (UK)	374	0.04
8	Univ Copenhagen (Denmark)	364	0.08
9	Massachusetts Gen Hosp (USA)	263	0.01
10	Kings Coll London (UK)	254	0.01

The ranking of institutions based on their contributions to Mendelian randomization research articles published between 2003 and 2023. It provides information about each institution, including its name, country, publication frequency, and centrality score.

### 3.7. Author analysis

**Figure 9.**
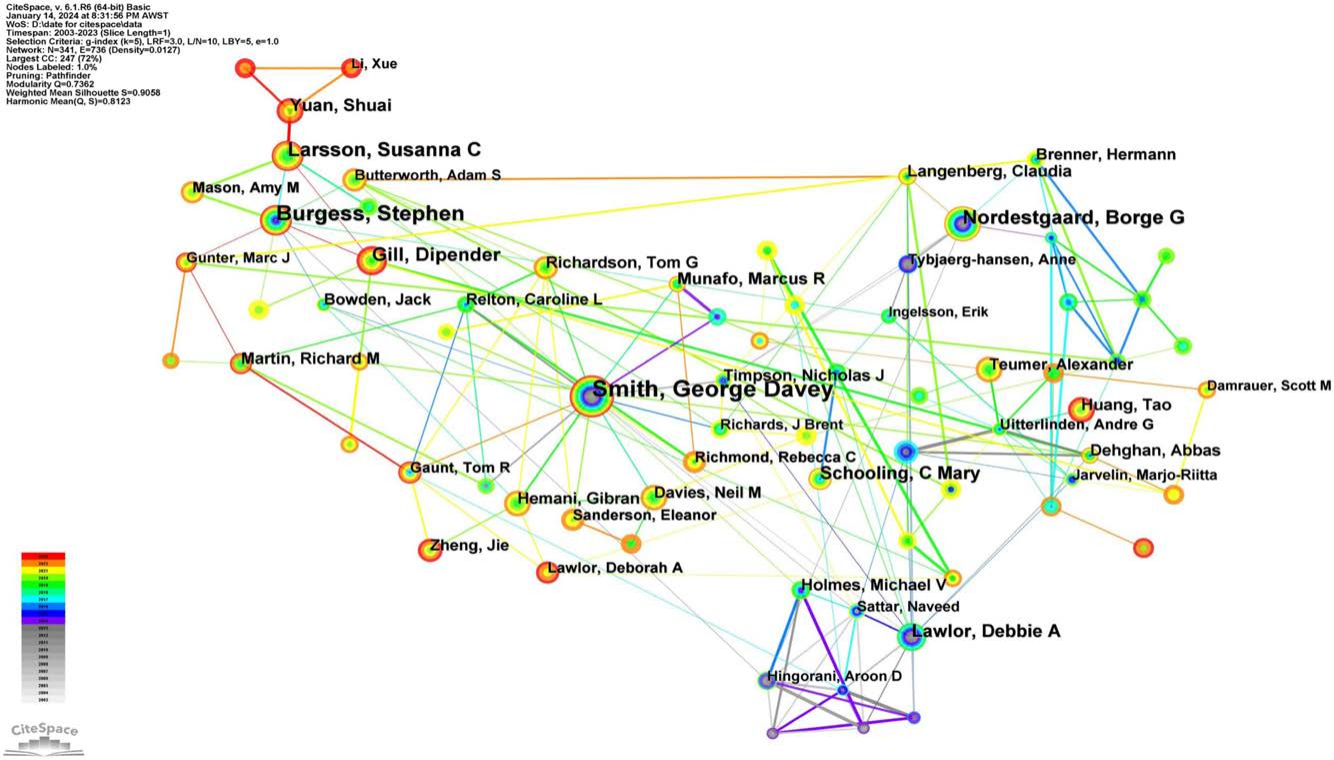
The network map of Core Author in Mendelian Randomization from 2003 to 2023. The size of the node indicates the author’s publication volume, and the lines depict the collaboration relationships between authors. There are 4 main author collaboration clusters, centered around Smith, George Davey; Burgess, Stephen; Nordestgaard, Borge G; and Debbie A Lawlor, respectively.

A total of 340 authors have contributed to the MR literature. Among them, 5 authors have each published over 100 papers. The top 10 authors were ranked by publication count, and their centrality scores were tabulated (Table 7). George Davey Smith leads with 264 publications, followed by Stephen Burgess (n = 264), Borge G Nordestgaard (n = 173), and Susanna C Larsson (n = 149). Applying Price law (N = 0.749 × √n_max), we identified 16 core authors in the MR field using the criterion of authors who have published 56 or more papers (16/340, 4.71%) and constructed a co-authorship network diagram of these core authors (Fig. 9). Nodes for Smith, George Davey; Stephen Burgess; and Nordestgaard, Borge G are larger due to their higher number of published articles. The figure displays 4 main author collaboration clusters, centered around Smith, George Davey; Burgess, Stephen; Nordestgaard, Borge G; and Debbie A Lawlor, respectively.

**Table 7:** Top 10 authors by frequency from 2003 to 2023.

Author	Frequency	Centrality	Institution
Smith, George Davey	461	0.13	Univ of Bristol (UK)
Burgess, Stephen	264	0.13	Univ of Cambridge (UK)
Nordestgaard, Borge G	173	0.07	Univ of Copenhagen (Denmark)
Larsson, Susanna C	149	0.07	Karolinska Institute (Sweden)
Gill, Dipender	129	0.05	Imperial College London (UK)
Schooling, C Mary	99	0.07	Univ of Hong Kong (China)
Lawlor, Debbie A	94	0.21	Univ of Bristol (UK)
Yuan, Shuai	80	0.06	Univ of Zhejiang (China)
Munafo, Marcus R	73	0.00	Univ of Bristol (UK)
Holmes, Michael V	64	0.04	University of Oxford (UK)

The top 10 authors ranked by the frequency of their contributions to Mendelian randomization research articles published from 2003 to 2023. It includes the author’s name, publication frequency, centrality score, and affiliated institution.

### 3.8. Co-cited reference analysis

**Figure 10.**
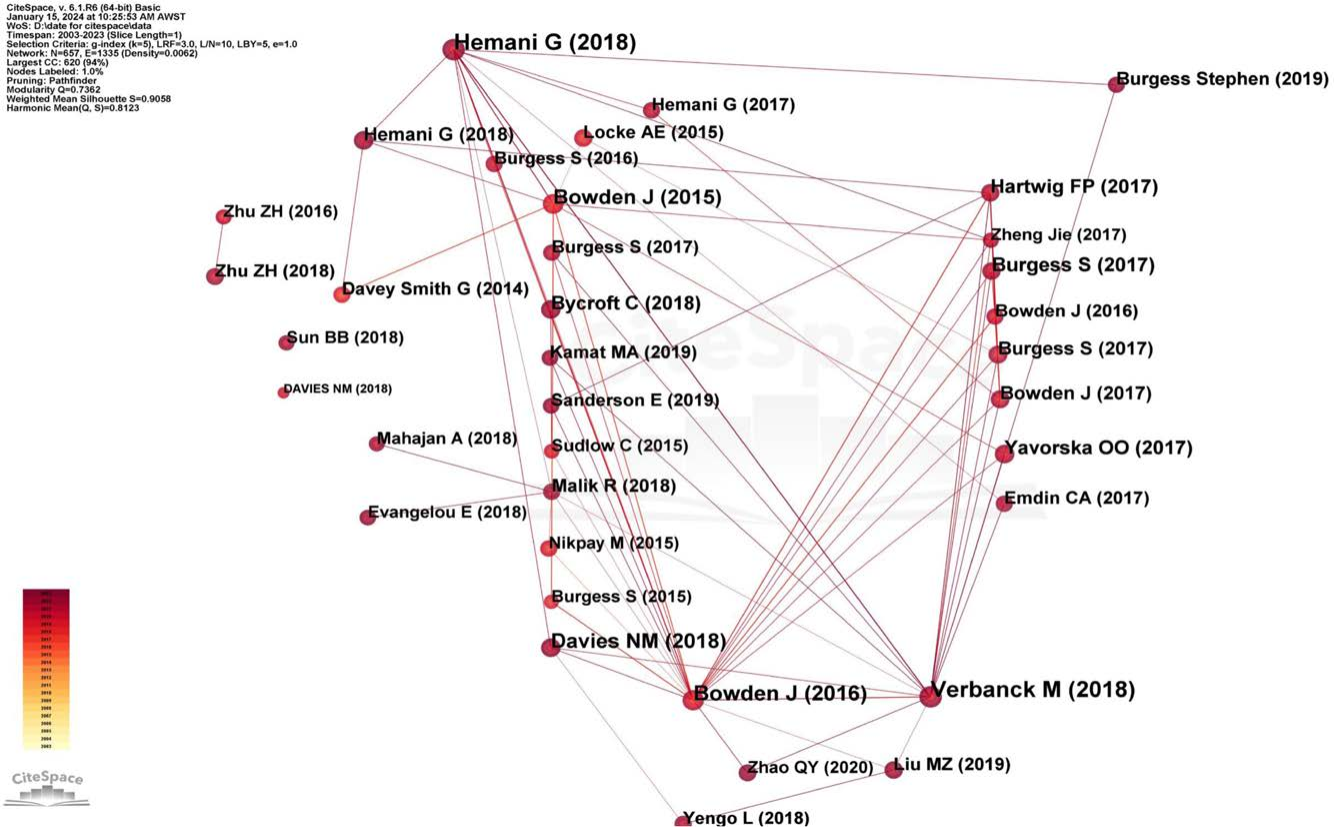
The network map of co-cited references in MR research from 2003 to 2023. The size of each node represents its co-citation frequency, while the links represent co-citation relationships among journals. MR = Mendelian randomization.

In our study, we pinpointed a total of 684 co-cited references. Of these, 649 publications registered co-citation frequencies below 200, 32 publications recorded frequencies between 200 and 700, and a distinct 3 publications boasted frequencies exceeding 1000. Table 8 lists the top 10 co-cited publications. Topping the chart is “ The MR-Base platform supports systematic causal inference across the human phenome “ by Hemani, Gibran, et al published in ELIFE, with an unparalleled co-citation frequency of 1625. This research team developed a platform that integrates a curated database of complete GWAS results (no restrictions according to statistical significance) with an application programming interface, web app and R packages that automate 2SMR. The software includes several sensitivity analyses for assessing the impact of horizontal pleiotropy and other violations of assumptions. These research ensures more rigorous application of hypothesis-driven analyses and allows millions of potential causal relationships to be efficiently evaluated in phenome-wide association studies.^[13]^ Following this, Verbanck, Marie et al published “Detection of widespread horizontal pleiotropy in causal relationships inferred from MR between complex traits and diseases” in NATURE GENETICS (n = 1620). In their contribution, the research ensemble introduced the MR pleiotropy residual sum and outlier (MR-PRESSO) methodology, adept at identifying and amending horizontal pleiotropy outliers in summary-level MR examinations. Their method’s prowess, the MR-PRESSO test, was meticulously gauged through expansive simulations and juxtaposed with other analogous methods.^[14]^ We curated the co-cited reference network graph (Fig. 10) using publications that met or surpassed a co-citation frequency of 200 times. The magnitude of each node mirrors its cited frequency. The most significant node corresponds to Hemani G et al publication (n = 1625), which establishes dense co-citation networks with numerous publications such as “Bowden J (2016),” “Verbanck M (2018),” “Davies NM (2018),” “Bycroft C (2018),” and others. “Verbanck M (2018),” “Burgess S (2017),” “Bowden J (2017),” and “Yavorska OO (2017)” demonstrate a frequent co-citation association. “Bowden J (2016)” is frequently co-cited with “Bowden J (2015),” “Malik R (2018),” “Zhao QY (2020),” and others.

**Table 8:** The top 10 co-cited references in Mendelian randomization research from 2003 to 2023.

Ranking	Frequency	Centrality	year	Publication
1	1625	0.15	2018	Hemani G, 2018, ELIFE, V7, P0, DOI 10.7554/eLife.34408
2	1620	0.11	2018	Verbanck M, 2018, NAT GENET, V50, P693, DOI 10.1038/s41588-018-0099-7
3	1039	0.41	2016	Bowden J, 2016, GENET EPIDEMIOL, V40, P304, DOI 10.1002/gepi.21965
4	793	0.13	2015	Bowden J, 2015, INT J EPIDEMIOL, V44, P512, DOI 10.1093/ije/dyv080
5	750	0.02	2018	Davies NM, 2018, BMJ-BRIT MED J, V362, P0, DOI 10.1136/bmj.k601
6	583	0.01	2017	Burgess S, 2017, EUR J EPIDEMIOL, V32, P377, DOI 10.1007/s10654-017-0255-x
7	531	0.01	2017	Yavorska OO, 2017, INT J EPIDEMIOL, V46, P1734, DOI 10.1093/ije/dyx034
8	503	0.02	2017	Hartwig FP, 2017, INT J EPIDEMIOL, V46, P1985, DOI 10.1093/ije/dyx102
9	478	0.06	2018	Bycroft C, 2018, NATURE, V562, P203, DOI 10.1038/s41586-018-0579-z
10	393	0.02	2018	Hemani G, 2018, HUM MOL GENET, V27, PR195, DOI 10.1093/hmg/ddy163

The top 10 co-cited references found within Mendelian randomization research articles published between 2003 and 2023. Each entry includes the ranking, co-cited reference, and the corresponding count of co-citations. The references are presented in descending order based on the number of co-citations.

### 3.9. Highly cited publication analysis

From the 7801 articles analyzed, we selected 987 publications with citation counts exceeding 50.

#### 3.9.1. Publication venues of highly cited papers

Table 9 presents the top 10 countries/regions ranked by their number of highly cited publications. The countries/regions include ENGLAND (n = 563), USA (n = 438), GERMANY (n = 180), NETHERLANDS (n = 179), AUSTRALIA (n = 161), SWEDEN (n = 154), DENMARK (n = 151), CANADA (n = 131), SCOTLAND (n = 124), and ITALY (n = 118). This analysis reveals that the top 10 highly cited papers predominantly originate from developed countries. This trend corresponds closely with the ranking of countries by publication volume in the MR domain. England is distinguished by having the highest number of highly cited papers, demonstrating the greatest centrality.

**Table 9:** The top 10 countries with highly cited papers in Mendelian randomization research.

Rank	Country	Centrality	Frequency
1	England	0.12	563
2	USA	0.01	438
3	Germany	0.01	180
4	Netherlands	0.02	179
5	Australia	0.02	161
6	Sweden	0.01	154
7	Denmark	0.01	151
8	Canada	0.02	131
9	Scotland	0.03	124
10	Italy	0.05	118

The top 10 countries with highly cited papers in Mendelian randomization research. The ranking is based on the frequency of highly cited papers published by each country during this period, and it also includes the centrality score for each country.

#### 3.9.2. Publication institutions of highly cited papers

**Figure 11.**
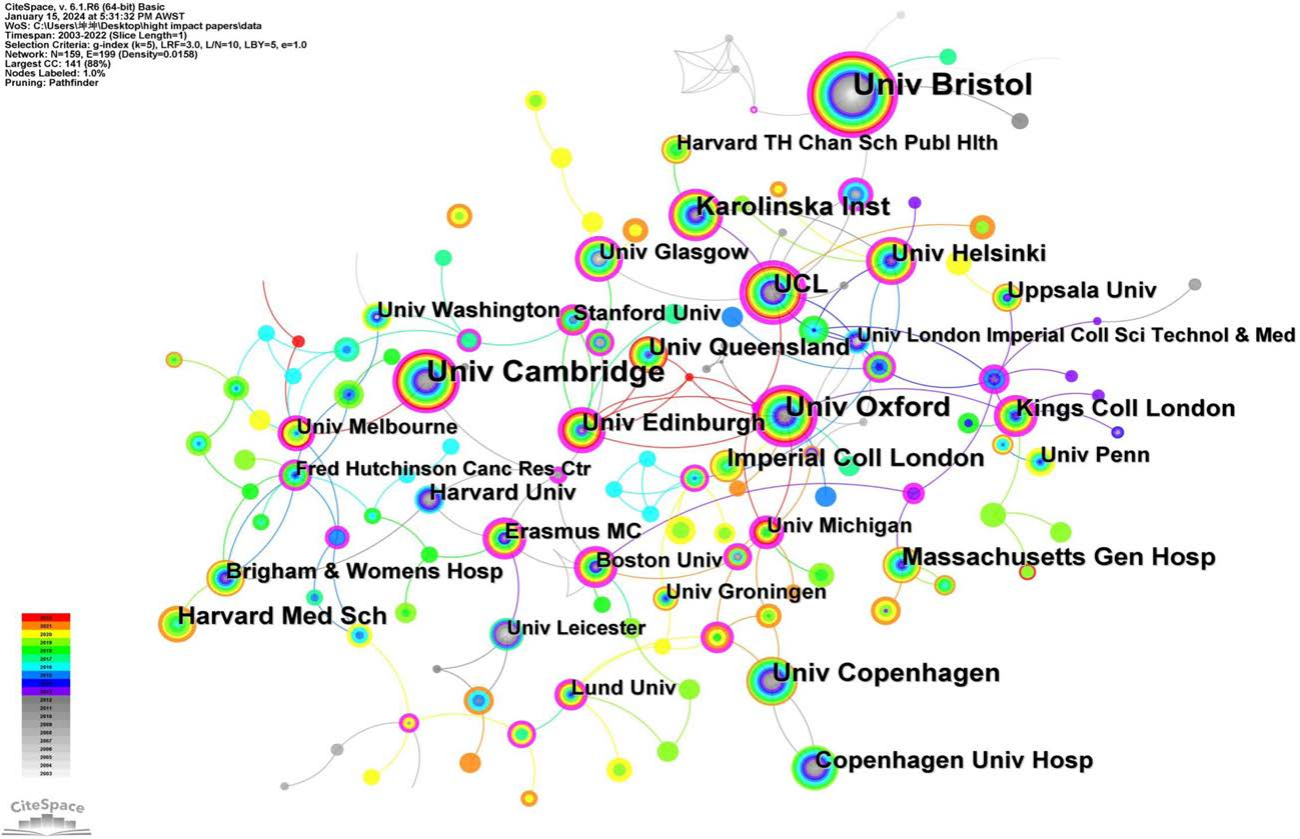
The network map of institution collaborations of highly cited papers in MR research. The collaborative network among these institutions based on their publications of highly cited papers, where nodes represent the number of highly cited papers and links indicate collaborations between institutions. MR = Mendelian randomization.

Table 10 displays the top 10 institutions with the highest number of highly cited papers. The institutions are Univ Bristol, Univ Cambridge, Univ Oxford, Univ Copenhagen, UCL, Karolinska Inst, Harvard Med Sch, Massachusetts Gen Hosp, Imperial Coll London, and Kings Coll London. Notably, of these institutions, 6 are from the UK, 2 from the United States, 1 from Sweden, and 1 from Denmark. Figure 11 illustrates the collaboration network among these institutions, based on their contributions to highly cited papers. Here, nodes represent the count of highly cited papers, while links highlight collaborations between institutions. Univ Bristol, possessing the leading count of highly cited papers (n = 271), is identified as the most substantial node.

**Table 10:** The top 10 institutions with highly cited papers in Mendelian randomization research.

Rank	institution	Centrality	Frequency
1	Univ Bristol	0.24	271
2	Univ Cambridge	0.16	205
3	Univ Oxford	0.33	139
4	Univ Copenhagen	0.02	100
5	UCL	0.45	94
6	Karolinska Inst	0.12	92
7	Harvard Med Sch	0.00	78
8	Massachusetts Gen Hosp	0.04	78
9	Imperial Coll London	0.00	75
10	Kings Coll London	0.25	71

The leading 10 institutions with highly cited papers in Mendelian randomization research. The ranking is determined by the frequency of highly cited papers published by each institution during this period, and it also includes the centrality score for each institution.

#### 3.9.3. Publication authors of highly cited papers

**Figure 12.**
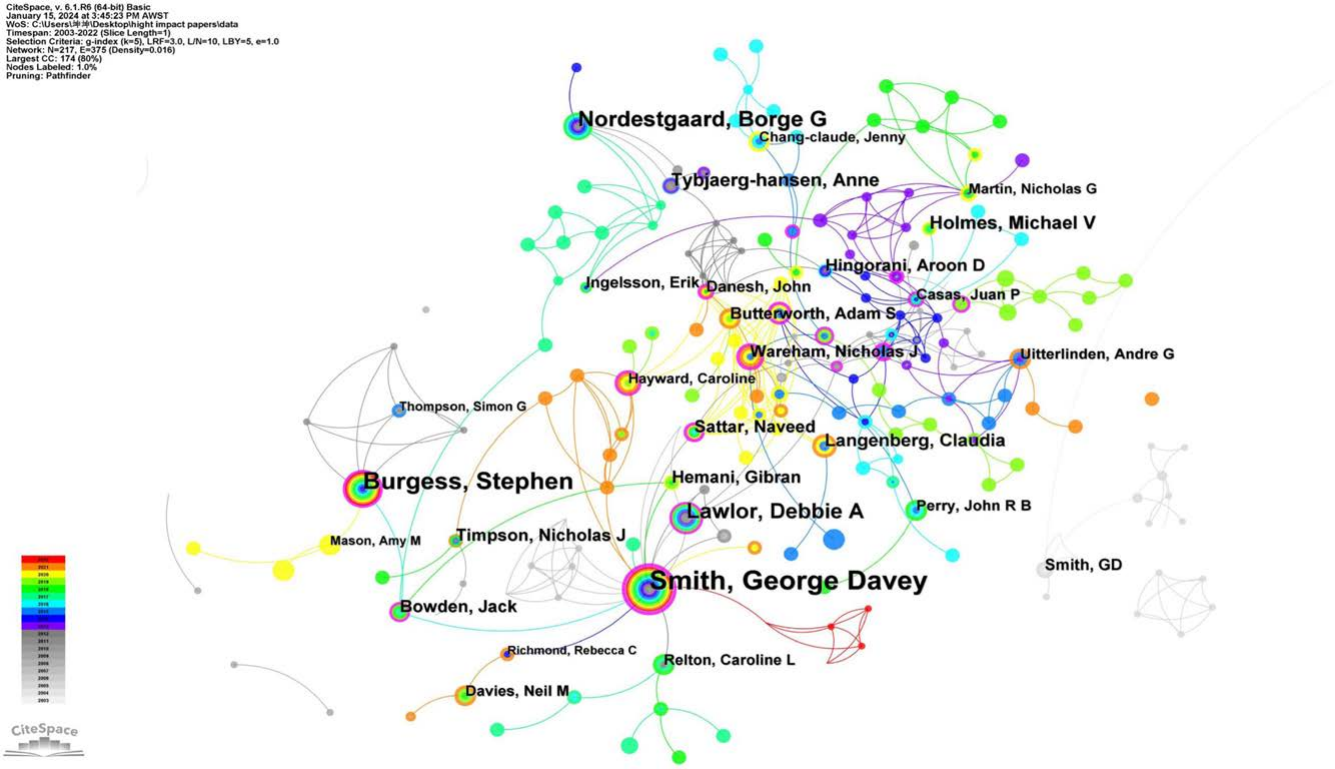
The network map of authors of highly cited papers in MR research. The collaborative network among authors who have published highly cited papers. Nodes represent the number of highly cited papers published by authors and links indicate collaborations between authors.

Table 11 enumerates the top 10 authors with the most significant contributions to highly cited papers. Leading the chart is George Davey Smith (n = 156), followed by Stephen Burgess (n = 84), Nordestgaard Borge G (n = 57), and Debbie A Lawlor (n = 47). Not only does George Davey Smith lead in contributions to highly cited papers, but he also has the highest centrality score (Centrality = 0.53). Figure 12 depicts the collaboration network among authors recognized for their highly cited papers. Within this network, George Davey Smith is the central figure, frequently collaborating with authors such as Lawlor Debbie A, Sattar Naveed, and Relton Caroline, among others. Meanwhile, Burgess and Stephen have formed a collaboration nexus with researchers such as Bowden Jack, Mason, Amy M, Tompson Simon G, and several others.

**Table 11:** The top 10 authors with highly cited papers in Mendelian randomization research.

Rank	Authors	Centrality	Frequency
1	Smith, George Davey	0.53	156
2	Burgess, Stephen	0.10	84
3	Nordestgaard, Borge G	0.05	57
4	Lawlor, Debbie A	0.13	47
5	Holmes, Michael V	0.00	27
6	Tybjaerg-Hansen, Anne	0.07	25
7	Langenberg, Claudia	0.01	23
8	Timpson, Nicholas J	0.04	21
9	Hemani, Gibran	0.00	21
10	Bowden, Jack	0.18	20

The top 10 authors with highly cited papers in Mendelian randomization research. The ranking is determined by the frequency of highly cited papers authored by each individual during this period, and it also includes the centrality score for each author.

## 4. Discussion

In this study, a bibliometric analysis was conducted on MR literature from 2003 to 2023 using CiteSpace, which was then compared with the analysis by Jianguo Xu et al covering MR literature from 2003 to 2020. This study also explored potential reasons for the observed disparities in the data.

### 4.1. Analysis of publication volume trends

The evolution of MR was segmented into 3 stages according to annual publication trends: a slow growth period (2003–2011), a rapid growth period (2012–2017), and an explosive growth period (2018–2022). The inaugural paper, “Mendelian randomization: can genetic epidemiology contribute to understanding environmental determinants of disease?” authored by George Davey Smith and Shah Ebrahim, emphasizes the potent explanatory prowess of MR in discerning the causal effects of exposure factors on disease risk as compared to numerous traditional observational epidemiology methods.^[3]^ The slow growth phase is possibly attributed to the nascent stages of in-depth research and the MR method not yet gaining widespread recognition in hands-on epidemiology, despite its proposed implementation in the domain. Furthermore, advancements in MR research were notably hindered by the limitations of the GWAS databases at the time and the constraints of computational technology. During the rapid growth stage, there was a surge from 107 papers in 2012 to 378 papers in 2017. Jianguo Xu et al attributed the swift increase in MR-related publications to milestones such as the completion of the Human Genome Project, the maturation and evolution of genome-wide association studies, and scholarly reflections on the constraints of randomized controlled trials.^[15]^ Concurrently, the rapid advancement of computer technology has enabled the processing of large genetic datasets and the conducting of more complex analyses. The explosive growth period, spanning 2020 to 2022, saw a notable average annual increase of 387 papers. Several factors can be ascribed to this pronounced increase in publications over these years. Firstly, the widespread availability of extensive GWAS datasets has provided a rich resource of genetic variants for use in MR studies. Secondly, the global COVID-19 pandemic intensified the research focus on exposure determinants and disease outcomes related to COVID-19. Moreover, increased funding was directed towards public health research, driving advancements in MR studies. For instance, Rao et al utilized MR to identify diseases/traits potentially bearing a causal relationship with ACE2 expression in the lung, influencing vulnerability to the virus.^[16]^ Thirdly, over time, MR has gained widespread recognition as a robust research methodology for evaluating causal relationships between exposure factors and outcomes in the academic community. As a result, an increasing number of researchers have focused on the MR field, leading to a significant increase in relevant publications. The findings highlight that, given its distinctive advantages over randomized controlled trials, such as addressing reverse causality and confounding variables, the rising prominence of MR studies in the academic community is evident. This trend indicates an anticipated increase in related research in the near future.

### 4.2. Keyword analysis

Keywords in the MR literature from 2003 to 2023 were analyzed using CiteSpace. In this keyword co-occurrence analysis, cardiovascular disease (CVD) emerged as the primary research hotspot. This included terms such as Blood pressure, Coronary heart disease, Coronary artery disease, Myocardial infarction, Heart disease, Atrial fibrillation, and Hypertension. Metabolic syndrome was identified as the next major research area, featuring keywords such as Blood pressure, Body mass index, Obesity, Insulin resistance, Type 2 diabetes, Density lipoprotein cholesterol, and Hypertension. Biomarkers constituted the third significant area, encompassing keywords including Vitamin D, C-reactive protein (CRP), and Density lipoprotein cholesterol. Other notable research areas included Inflammation, AD, Cancer, and Age, among others.

CVD has been a central topic in MR research since 2003. Studies have primarily focused on exposure factors such as CRP, low-density lipoprotein cholesterol (LDL-C), and BMI. CRP, functioning as a biomarker, has been shown to increase the risk of hypertensive heart disease by 21%. However, studies found no link between serum CRP levels and the adjusted risk of other cardiovascular diseases, including myocardial infarction, coronary heart disease, heart failure, and atherosclerosis. Furthermore, evidence did not suggest reverse causation, implying that CVD does not affect CRP levels.^[17]^ Brian A. Ference and colleagues presented strong evidence indicating that LDL is not merely a risk marker but a causal factor in the development of Atherosclerotic Cardiovascular Disease.^[18]^ Susanna C. Larsson and her team conducted MR studies, identifying a significant relationship between a higher BMI, especially fat mass index, and an increased risk of aortic stenosis and other cardiovascular diseases.^[19]^

AD, a neurodegenerative condition, is characterized by steady cognitive decline, memory impairment, and changes in behavior and emotion. As current treatments are unable to halt or slow the disease’s progression, focusing on interventions targeting modifiable risk factors to reduce AD incidence is crucial. Emma L. Anderson and her team provided strong evidence suggesting an independent and causal role of intelligence in reducing AD risk. Consequently, cognitive training to enhance intelligence is considered a potential approach to lower AD risk.^[20]^ Additionally, decreased levels of 25-hydroxyvitamin D have been identified as a risk factor for AD. However, extended RCTs are necessary to ascertain the potential preventive impact of vitamin D supplementation on AD.^[21]^ Type 2 diabetes (T2DM), a long-term metabolic disorder, is characterized by insulin resistance and insufficient insulin production, and is commonly observed in middle-aged or older adults. Significant research has focused on identifying exposure factors associated with T2DM risk. For example, Luca A. Lotta and his team discovered that polymorphisms increasing BCAA levels, identified through a genome-wide approach, were associated with an increased risk of T2DM.^[22]^ Wang, TG’s team underscored that genetically reduced birthweight is linked to a higher risk of type 2 diabetes.^[23]^

The top 20 keywords from 2003 to 2023 were compared with those from 2003 to 2020.^[15]^ It was found that cardiovascular diseases, metabolic diseases, inflammation, biomarkers, cancer, and AD remained consistent topics of interest in both datasets. Despite the different time spans, these research hotspots consistently attracted attention, highlighting their significance in guiding clinical practices and preventive measures. However, keywords such as Smoking and Alcohol from the 2003 to 2020 dataset were not present in the top 20 keywords of the 2003 to 2023 set. Instead, terms such as mortality and age emerged in the 2003 to 2023 dataset. This shift in keywords indicates evolving research priorities and trends within the MR field. Possible factors include the ongoing updates and refinements of the GWAS database, shifting focus from specific behavioral risk factors (such as smoking and alcohol consumption) to associations with more complex health outcomes (like mortality). Thomas Lawler and colleagues’ research found no support for causal associations between 25-hydroxyvitamin D levels and the risk of cancer incidence or mortality.^[24]^ However, another study identified a causal relationship between 25 (OH)D concentrations and mortality in individuals with low vitamin D status.^[25]^

The ongoing intensification of global population aging, along with in-depth research into age-related diseases, represents one of the possible contributing factors. Stephen, Burgess, et al demonstrate that the multivariable MR method reveals an inverse direct causal effect of age at menarche on the risk of breast cancer (independent of BMI), and a positive indirect effect via BMI.^[26]^ Xiong, A. identified no evidence of a causal role for serum uric acid in bone-related outcomes, although strong associations were observed in an observational analysis within a population of postmenopausal women and elderly men.^[27]^ It is essential to recognize that these shifts do not diminish the importance of earlier research topics; rather, they reflect evolving areas of focus within the MR research field.

In the keyword burst analysis spanning 2020 to 2023, we observed a notable shift in the research focus of the MR field. Four keywords: “coffee consumption,” “obstructive sleep apnea,” “psychiatric disorder,” and “major depressive disorder,” have emerged as prominent. Over the past 3 years, coffee consumption has garnered significant attention from researchers. A study led by Yunyang Deng suggested that although genetically predicted alcohol use and consumption are risk factors for colorectal cancer, genetically predicted coffee consumption appears to provide protective effects against colorectal cancer.^[28]^ In another study by Yudong Wei team, both observational and MR analyses indicated that coffee intake, particularly instant coffee, might be associated with a reduction in telomere length, while filtered coffee exhibited no such relationship.^[29]^ Meanwhile, Zhizhong Zhang team provided evidence indicating that coffee consumption could increase the risk of intracranial aneurysm and related hemorrhage. Consequently, individuals at high risk for intracranial aneurysm and related hemorrhage might consider moderating their coffee consumption.^[30]^

OSA is a serious sleep disorder characterized by repeated interruptions in nighttime breathing due to upper airway blockage. This condition results in intermittent hypoxia, systemic inflammation, and activation of the sympathetic nervous system, potentially leading to severe complications. Xiao-Ling Gao and colleagues employed a two-sample MR analysis to demonstrate a causal link between an elevated risk of OSAS and an increased likelihood of breast cancer. This connection might be attributed to the hypoxia induced by OSAS, which plays a role in the onset and progression of breast cancer.^[31]^ Prior observational studies have indicated a potential connection between OSA and atrial fibrillation. Building upon this, Weiqi Chen team conducted an MR analysis employing 5 SNPs as instrumental variables, establishing a causal relationship between OSA and an increased risk of atrial fibrillation.^[32]^

Wu et al underscored a potential bidirectional causal relationship between psychiatric disorders and Parkinson disease (PD). Their study suggests that psychiatric disorders and traits may influence the risk of developing PD, and conversely, PD may also contribute to the risk of developing psychiatric disorders.^[33]^ Karmel W. Choi and colleagues found that physical activity might confer protection against the risk of major depressive disorder. Their results also support the notion that enhancing physical activity could be an effective strategy for depression prevention.^[34]^

Comparing keyword burst analysis results from 2020 to 2023 with those from 2003 to 2020, Jianguo Xu et al identified future research hotspots, including LDL-C, biomarkers, coffee, oxidative stress, and telomere length from 2003 to 2020.^[15]^ Our analysis of the data from 2020 to 2023 corroborated these findings. While only “biomarkers” ranked among the top 20 in frequency (n = 75), LDL-C (n = 11), coffee (n = 15), oxidative stress (n = 68), and telomere length (n = 32) also appeared notably. This demonstrates that LDL-C, coffee, oxidative stress, and telomere length remain focal points in the MR field and active research topics from 2020 to 2023. Additionally, we observed that OSA, psychiatric disorder, major depressive disorder, and coffee consumption have emerged as prominent research topics in recent years. Consequently, we anticipate that future MR research hotspots will encompass biomarkers, LDL-C, coffee consumption, oxidative stress, telomere length, OSA, psychiatric disorder, and major depressive disorder.

### 4.3. Keywords clustering

By identifying co-occurrence patterns and similarities among keywords, we grouped similar topics or research areas into clusters. This approach elucidates the close associations between different keywords, thereby assisting researchers in comprehending the structure and developmental trends within a literature domain. We identified 16 distinct clusters (refer to Fig. 4). Upon comparing our clustering results with those of Jianguo Xu et al, who analyzed keyword clustering in the MR field from 2003 to 2020, we observed substantial similarities. Nonetheless, the themes for the period from 2003 to 2023 also accentuated ischemic stroke (IS), birth weight, and metabolic syndrome. In the past 3 years, there has been a surge in publications concerning IS, indicating increased attention to MR’s role in IS research. For example, Biyan Wang et al used a two-sample MR analysis, drawing from summary genetic data from both East Asian and European populations, to examine the potential causal effect of genetically determined IgG N-glycosylation on IS. Interestingly, their MR analysis did not produce strong evidence of a causal link between genetically determined IgG N-glycosylation and IS, contrasting findings from earlier observational studies.^[35]^ Similarly, Renjie Liu et al performed a two-sample MR analysis to assess the relationship between ischemic stroke and cerebral microbleeds. Their research substantiated a causal association, suggesting that IS is directly linked to an increased incidence of CMBs.^[36]^ Research has also explored the potential adverse prognostic risks in IS patients due to rheumatoid factor and the increased risk of Frozen Shoulder in individuals with IS. Birth weight may act as an exposure factor associated with an increased risk of developing NAFLD. For instance, research has demonstrated a causal relationship between lower birth weight and NAFLD, irrespective of subsequent fat accumulation.^[37]^ Additionally, birth weight can also be an outcome influenced by exposure factors. For example, increased testosterone levels in females within the general population have been associated with the low birth weight of their offspring.^[38]^ Other research avenues have investigated the causal relationship between birth weight and mental health, osteoporosis, among others.

### 4.4. Journal and co-cited journal analysis

Between January 1, 2003, and July 8, 2023, a total of 7801 academic articles pertaining to MR were published in 1235 academic journals. Among these journals, the International Journal of Epidemiology had the highest number of publications, totaling 292 articles. The journal Circulation has the highest impact factor. This is followed by Nature Communications (IF = 16.6, n = 132) and BMC Medicine (IF = 9.3, n = 95).

There are notable distinctions between our research findings on journals from 2003 to 2023 and those of Jianguo Xu et al from 2003 to 2020. In the past 3 years, the journal with the most significant increase in publication volume is the International Journal of Epidemiology, which saw an increase of 192 articles from 2003 to 2023 compared to 2003 to 2020. This increase is likely attributable to the growing emphasis on MR methods in epidemiology and public health research. Additionally, emerging journals include Frontiers in Genetics, Genetic Epidemiology, Nutrients, Frontiers in Endocrinology, Circulation, and BMC Medicine. The potential reason for this phenomenon may be the shift in research focus and trends. During the period from 2003 to 2020, the MR research field emphasized epidemiological studies, particularly during the COVID-19 pandemic, which might have attracted more research and submissions related to infectious diseases. Consequently, the International Journal of Epidemiology published the highest number of articles from 2003 to 2020 [15]. However, from 2020 to 2023, the focus of MR research gradually shifted towards genetic and endocrinology studies, leading to Frontiers in Genetics and Frontiers in Endocrinology taking the second and eighth positions, respectively, in publication volume during this period, becoming emerging journals, while journals like the International Journal of Epidemiology, focusing on epidemiology, still maintained a high volume of publications.

Among the co-cited journals, the top 10 boast an average impact factor of 52.65, indicating a preference among researchers for utilizing literature from high-impact factor journals to substantiate their arguments in scholarly writing. Nature Communications (n = 2962, IF = 16.6) and Nature (n = 3517, IF = 64.8) are pivotal journals in the field of MR research, exerting considerable influence as foundational sources of knowledge at the research forefront. Consequently, when selecting journals for submission, researchers may give priority to these journals. Regarding co-cited journals, the top 10 journals with the highest citation frequency from 2003 to 2023 exhibit similarities to the data from 2003 to 2020, demonstrating their consistent publication of high-quality research outputs in the MR field.

In the dual overlay map of journals, the starting position of citation trajectories in this study is predominantly influenced by literature from the molecular, biology, immunology, medical, and clinical fields, dominating the citation base map. However, the ending position of citation trajectories appears to be influenced by activities in the psychology, education, and health domains. The position in the cited base map is primarily determined by literature in the fields of molecular, biology, genetics, nursing, and medicine, which collectively form the knowledge foundation of MR research.

### 4.5. Country/region/institution analysis

In the visualization of country/institution collaboration, the top 3 countries were England (n = 2039), China (n = 1203), and the USA (n = 1098). This outcome is slightly different from the data spanning 2003 to 2020 presented by Jianguo Xu et al. Their report showed that in this period, the UK contributed 852 articles, the USA followed with 725 articles, and China produced 404 articles [15]. The shift could be attributed to changes in China’s scientific policies, such as the “13th Five-Year Plan” and the National Key R&D Program, which prioritize biomedical and biotechnological research, thereby encouraging studies including MR. Moreover, China possesses a larger researcher population compared to other countries, establishing a foundation for the rapid increase in MR research output. Additionally, our analysis of the national/regional cooperation network indicates strong collaborations among countries, attributable to MR’s reliance on large-scale genomic data and disease risk data, typically obtained from multiple countries and regions. Meanwhile, China engages in collaboration with only a few countries, such as the USA and Singapore. Despite its substantial output, the lack of international cooperation could result in various disadvantages, including information and research insularity, which may impede MR’s development in China. Therefore, it would be advantageous for Chinese researchers to actively foster ties with the broader international academic community, thereby fostering deeper insights into the MR field.

The University of Bristol (UK) holds the first rank with 1084 publications, thereby establishing itself as a leader in the field. The university’s research encompasses multiple domains, and its findings are widely published in high-impact academic journals, significantly influencing many research areas. In CiteSpace, Betweenness Centrality serves as a metric in network analysis that measures the role of a node as a connector within the network. Institutions with high Betweenness Centrality are considered to possess considerable influence and standing in the academic world. We noted that the Univ Bristol, Univ Oxford, and Univ Cambridge not only boast impressive publication numbers but also exhibit significant intermediary centrality, indicating close cooperative relationships between them, potentially due to their shared location in the UK. This implies that researchers from different institutions may favor collaborating with domestic institutions. In the global research output of the MR field, although China ranks second overall in terms of paper production, not a single Chinese institution appears in the top 10 global institutional rankings. This suggests that MR research in China is relatively dispersed, involving multiple academic institutions. In contrast, MR research in other countries appears more concentrated in a limited number of institutions, which might explain their prominent positions in global rankings. Such distributional differences could arise from various factors, including the allocation of research resources in different countries, structural differences in academic cooperation networks, and variations in choices for publishing research outcomes.

Upon comparing institutional rankings between 2003 to 2023 and 2003 to 2020 [15], it is evident that the top 10 institutions in terms of publication volume have remained relatively consistent. Both the University of Bristol and the University of Cambridge retain their top 2 spots across both periods. However, University College London (UK), which ranked 6th in publication volume between 2003 and 2020, failed to secure a position in the top 10 in the 2003 to 2023 period. This discrepancy could be attributed to shifts in their research focus and funding allocation.

### 4.6. Author analysis

In the analysis of authors contributing to MR-related literature, George Davey Smith ranks first with 461 publications. He has made significant contributions in areas such as MR, public health, health inequalities, epidemiological methods, and causal inference. For instance, George Davey Smith published the first paper in the field of MR, stating that associations between modifiable exposures and disease seen in observational epidemiology can sometimes be confounded and thus misleading. However, the association between a disease and a polymorphism that mimics the biological link between a proposed exposure and disease is generally not susceptible to the reverse causation or confounding that may distort interpretations of conventional observational studies. At the same time, the limitations of the MR method were articulated.^[3]^ In his publication “Tackling Health Inequities,” Davey Smith discusses global health inequity and emphasizes the impact of social injustice on health. He synthesized evidence from various sources to highlight the links between economic, social, and bodily well-being within and across countries.^[39]^ Burgess, Stephen (n = 264, Centrality = 0.13, UK), George Davey Smith (n = 461, Centrality = 0.13, UK), Gill, Dipender (n = 129, Centrality = 0.05, UK), Nordestgaard, Borge G (n = 173, Centrality = 0.07, Denmark), and Larsson, Susanna C (n = 149, Centrality = 0.07, Sweden) not only exhibit a high volume of publications but also possess significant centrality values. This indicates that these scholars not only have numerous publications in the MR field but also play a crucial bridging and connecting role in the academic network structure. Figure 9 displays 4 main collaboration groups centered around George Davey Smith, Stephen Burgess, Borge G Nordestgaard, and Debbie A Lawlor. In these groups, most authors generally belong to the same institution. For instance, the group centered around Stephen Burgess primarily includes researchers from the University of Cambridge. However, this fact does not hinder extensive cross-institutional collaboration among group members. Our comparative analysis of the publication data from 2003 to 2020 [1] revealed no significant distinction in the top 10 authors in terms of publication volume between this period and the current data.

### 4.7. Co-cited reference analysis

Co-cited references are those that appear together in the citations of other publications.^[40]^ We discovered that the average impact factor of the top 5 co-cited publications is 31.2, suggesting that researchers tend to cite literature from high-impact factor journals to provide a robust theoretical foundation for their studies. Upon comparing the top 10 co-cited publications from 2003 to 2023 with those from 2003 to 2020 [15], we made 2 main observations. First, the co-citation counts for the latter period were consistently lower than those in the former. This phenomenon can be attributed to the explosive growth of MR-related literature from 2020 to 2023. Second, the paper titled “Mendelian Randomization: Can Genetic Epidemiology Contribute to Understanding Environmental Determinants of Disease?” authored by Smith GD and Ebrahim S in the International Journal of Epidemiology emerged as the most cited for the period 2003 to 2020. In contrast, from 2003 to 2023, the most cited paper shifted to “The MR-Base Platform Supports Systematic Causal Inference across the Human Phenome” by Hemani G et al, published in ELIFE in 2018. The prominence of the 2003 paper by Smith GD and Ebrahim S is attributable to its pioneering role in systematically introducing the principles, applications, and significance of MR methods. Consequently, it has been frequently cited by subsequent researchers in their studies to strengthen their theoretical frameworks and knowledge base.^[3]^ However, accessing genetic data for MR can be challenging, and MR methods, which are evolving rapidly, can pose implementation difficulties for non-specialists. To address the need for more systematic curation and application of complete GWAS summary data and MR methods, Hemani G et al developed MR-Base, a platform that integrates a database of thousands of GWAS summary datasets with a web interface and R packages for automated causal inference through MR. This innovative approach not only automates the process of MR but also significantly accelerates research outcomes and enhances the reliability of causal inference in MR studies.^[13]^ This rendered Hemani G et al paper an essential reference for MR researchers, resulting in its frequent citation from 2020 to 2023. Moreover, since Jianguo Xu et al bibliometric analysis of MR literature only included data up to 2020 [15] and Hemani G et alpaper was published in 2018, its recent publication date may have contributed to a lower co-citation count in the previous analysis.

### 4.8. Highly cited publication analysis

From the 7801 articles spanning 2003 to 2023, we selected 987 highly cited papers, each garnering more than 50 citations, for an analysis of high-citation papers. The analysis of highly cited papers provides deeper insight into research focus and trends in the MR field and offers researchers dependable references. Additionally, this approach evaluates the research capabilities and contributions of countries, institutions, and authors in this domain, hinting at potential collaborations. All of the top 10 countries contributing the most highly cited papers are developed nations. This trend is likely reflective of their substantial investments in the MR field, both in terms of funding and research workforce, leading to high-quality research outputs. Moreover, these countries often foster collaborations with peers globally, further advancing MR research. While China ranks second in publication volume (n = 2543), it ranks 12th for highly cited papers (n = 96), which is significantly behind the leading England (n = 563). Several reasons could explain this disparity. Many Chinese contributions might not reach the caliber of highly cited works. Additionally, the pioneering efforts in MR research originated from international scholars, granting them a head start in the field. Therefore, promoting international collaborations and exchanges could elevate China’s standing in the realm of impactful MR research. The institutions that produced the most highly cited papers all originated from developed nations, including the UK, the US, Sweden, and Denmark. The University of Bristol leads this tally, reflecting the substantial contributions from affiliated authors in the MR sector, such as George Davey Smith and Deborah A. Lawlor. In analyzing collaborations, we discern tight-knit networks among many institutions. For instance, the University of Oxford notably collaborates with the University of Michigan and Imperial College London, while UCL engages extensively with the Karolinska Institute and the University of Glasgow. Such collaborations undoubtedly contribute to growth in MR research. George Davey Smith, from the University of Bristol, emerges as a standout contributor to highly cited papers, signifying his influential role in the MR landscape. Intriguingly, only 5 authors who rank in the top 10 by publication volume for 2003 to 2023 also appear in the top 10 for highly cited paper contributions. This underscores that the frequency of citations of highly cited works does not solely depend on the author’s publication count but also hinges on research focus and paper quality. Consequently, relying solely on publication volume as a metric for gauging an author’s impact is insufficient. Upon examining the collaboration patterns of authors contributing to highly cited papers, distinct groups emerge. Notably, the main collaboration networks are centered around individuals such as George Davey Smith, Stephen Burgess, and Jack Bowden. Typically, authors within each cluster tend to be affiliated with a common institution. For instance, the George Davey Smith-centric group predominantly features authors from the University of Bristol, while the Stephen Burgess-centric group mainly comprises affiliates of the University of Cambridge. These patterns suggest intensive collaborations within these groups and their respective institutions. However, a broader examination of the collaboration network reveals their interconnections with international authors.

### 4.9. Limitations

Our study does have certain limitations. We based our analysis on the Web of Science Core Collection database, which houses a vast array of high-quality publications. However, it might not encompass all pertinent literature. Moreover, this database primarily focuses on English-language literature, potentially excluding other significant works in different languages. In the examination of highly cited papers, some recently published articles might not have gathered a high citation count yet, but they could become highly cited in the future.

## 5. Conclusion

This study employed CiteSpace to analyze literature related to MR research from January 2003 to July 2023. During this period, a rapid growth in the number of publications related to MR was observed. Research hotspots centered around cardiovascular diseases, metabolic diseases, biomarkers, inflammation, AD, cancer, and aging. Potential future research areas may include biomarkers, LDL-C, coffee consumption, oxidative stress, telomere length, OSA, psychiatric disorders, and major depressive disorder. The goal of this analysis was to discern changes in research hotspots over various time frames and to understand the shifts in focus and significance of MR research. This analysis provides valuable insights for future MR research directions.

## Acknowledgments

We would like to sincerely thank the team led by Jianguo Xu for their contributions to bibliometrics in the field of MR. Our heartfelt appreciation also goes to the reviewers for their valuable feedback and suggestions.

## Author contributions

**Conceptualization:** Zhengyu Qian, Kaijie Lin, Yue Wang, Zhikun Su, Tianyao Zhang.

**Data curation:** Zhengyu Qian, Kaijie Lin, Yue Wang, Zhikun Su.

**Formal analysis:** Zhengyu Qian, Kaijie Lin, Yue Wang, Zhikun Su.

**Investigation:** Xiaochu Wu.

**Methodology:** Kunyang He, Xiaochu Wu, Tianyao Zhang.

**Resources:** Tianyao Zhang.

**Software:** Kunyang He.

**Supervision:** Tianyao Zhang.

**Validation:** Xiaochu Wu, Tianyao Zhang.

**Visualization:** Kunyang He, Tianyao Zhang.

**Writing – original draft:** Kunyang He.

**Writing – review & editing:** Kunyang He, Xiaochu Wu, Tianyao Zhang.
